# Sarcopenia diagnosed by computed tomography predicts postoperative complications in advanced epithelial ovarian cancer

**DOI:** 10.1007/s40520-024-02901-9

**Published:** 2024-12-27

**Authors:** Shuyue Su, Rongrong Shao, Mengxiao Sun, Jingying Bai, Haote Jiang, Yuyang Zhang

**Affiliations:** 1 The First School of Medicine, School of Information and Engineering, Wenzhou Medical University, Wenzhou, 325000 China; 2 Department of Radiology, The First Affiliated Hospital of Wenzhou Medical University, Wenzhou, 325000 China; 3 Department of Obstetrics, The First Affiliated Hospital of Ningbo University, Ningbo, 315000 China; 4 Department of Ultrasound, The First Affiliated Hospital of Wenzhou Medical University, Wenzhou, 325000 China; 5 Department of Gynecology, The First Affiliated Hospital of Wenzhou Medical University, Wenzhou, 325000 China

**Keywords:** Ovarian cancer, Sarcopenia, Computed tomography (CT), Skeletal muscle area and index, Postoperative complications

## Abstract

**Background & aims:**

Surgery combined with chemotherapy remains the mainstay of treatment for advanced epithelial ovarian cancer. It is important to evaluate the occurrence of postoperative complications before operation and to prevent them. The purpose of this study is to investigate the role of sarcopenia diagnosed by CT scans in predicting postoperative complications in patients with ovarian cancer.

**Methods:**

Patients with PDS or IDS in the First Affiliated Hospital of Wenzhou Medical University from August 2017 to August 2022 were included. Sarcopenia was identified using CT scans at the T12 level. According to the Clavien-Dindo classification, postoperative complications are considered to have occurred in grades 2 and above. Logistic regression analysis was used to identify risk factors contributing to postoperative complications. *P* < 0.05 was considered statistically significant.

**Results:**

A total of 139 patients were included. Sarcopenia was observed in 24(17.27%) patients with advanced epithelial ovarian cancer. Compared to the non-sarcopenia group, the sarcopenia group had a higher incidence of postoperative complications (62.50% VS 42.61%, *p* = 0.032). Multivariate logistic analysis confirmed sarcopenia (OR = 3.241, *p* = 0.026), age over 65 years (OR = 3.296, *p* = 0.005), and intraoperative bleeding (OR = 1.002, *p* < 0.001) as independent predictors of postoperative complications in ovarian cancer.

**Discussion & conclusions:**

Sarcopenia diagnosed based on CT body composition analysis may serve as a potential predictor for postoperative complications. Further research is warranted to explore preventive strategies and interventions to improve outcomes in this population.

**Supplementary Information:**

The online version contains supplementary material available at 10.1007/s40520-024-02901-9.

## Introduction

### Ovarian cancer

Ovarian cancer (OC) imposes a substantial burden on women’s health in developed nations, including the United States, where it ranks as the fifth leading cause of cancer mortality, accounting for approximately 5% of cancer-related deaths [1]. Among the five major tissue types with varying origins, epithelial ovarian cancer (EOC) is the predominant pathological subtype, accounting for over 90% of ovarian cancers [2]. Due to its asymptomatic and insidious nature, epithelial ovarian cancer is often diagnosed at an advanced stage [3]. The current standard treatment for ovarian cancer involves primary cytoreductive surgery with complete resection of all visible lesions, followed by adjuvant platinum-based postoperative chemotherapy [4]. In case laparoscopic exploratory surgery confirms the non-feasibility of radical removal, neoadjuvant chemotherapy (NACT) is administered, followed by tumor reduction surgery after assessing surgical feasibility [5]. The 5-year survival rate for advanced epithelial ovarian cancer after surgical intervention is a mere 29%, in stark contrast to 92% rate in early stage [2]. The lowest mortality risk has been reported for the time to chemotherapy between 25 and 29 days after surgery, and the best survival benefit may be obtained when chemotherapy is started within 21 to 35 days after surgery [6]. Therefore, it is particularly important to reduce postoperative complications and shorten the time from surgery to chemotherapy in patients with advanced ovarian cancer.

### Postoperative complications

The incidence of postoperative complications after primary debulking surgery in ovarian cancer was reported to be 44% in previous studies, with a specific incidence of serious postoperative complications in advanced epithelial ovarian cancer reaching 22% [7, 8]. Therefore, it is imperative to comprehensively evaluate the risk of postoperative complications in surgical intervened patients with advanced epithelial ovarian cancer and implement appropriate preventive measures.

### Sarcopenia

The composition of lean body tissue has been identified as a most critical determinant of clinical regression of malignancy patients [9].Body mass index (BMI), derived from height and weight, as a commonly used indicator, fails to accurately reflects the true nutritional status of patients due to tumor-related weight loss being concealed by tumor burden and ascites. As a result, traditional nutritional indicators such as body mass index exhibit limited predictive performance for short-term postoperative outcome. Sarcopenia, characterized by progressive and generalized loss of skeletal muscle mass, has emerged as a prognostic factor in various malignancies such as gastric [10], liver [11] and renal cancer [12]. Previous investigations have predominantly focused on the association between sarcopenia and overall survival in ovarian cancer, with few studies examining its short-term impact on the postoperative period [13]. Accordingly, the aim of this study is to explore in depth the relationship between sarcopenia, estimated through cross-sectional computed tomography (CT) scans, and postoperative complications in patients with advanced epithelial ovarian cancer.

## Materials and methods

### Patient selection

The study population was comprised of patients with a confirmed diagnosis of ovarian cancer and underwent radical ovarian cancer surgery at the First Affiliated Hospital of Wenzhou Medical University between August 2017 and August 2022. It is remarkable that surgery consisted of primary debulking surgery (PDS) and interval debulking surgery (IDS). The patients satisfying the following inclusion were included: (i) initial and primary diagnosis of ovarian cancer; (ii) advanced surgical staging with confirmatory pathology report of epithelial ovarian cancer; (iii) chest or abdominal computed tomography (CT) scan either at our institution or an external facility, with accessible CT scan images. Patients were excluded if they met any of the following exclusion criteria: (i) presence of malignant tumors originating from other organs metastasizing to the ovary. (ii) primary ovarian cancer managed exclusively with chemotherapy alone. (iii) surgery procedure conducted at an external medical facility resulting in unavailability of clinically relevant information; (iv) absence of preoperative CT scans, rendering the collection of skeletal muscle content data unfeasible; (v) failure to comply with postoperative medical advice, resulting in incomplete data on short-term postoperative complications. In order to minimize potential bias, surgical interventions were exclusively by doctors holding the title of associate chief physician or above, with a minimum of 200 surgical experiences, while adhering to National Comprehensive Cancer Network (NCCN) guidelines to ensure reasonable standardization. This study was approved by the Research Ethics Committee of the First Affiliated Hospital of Wenzhou Medical University and was exempted from signing informed consent forms (KY2023-R288).

### Data collection

Data collection encompassed three key areas of patient information: (1) preoperative date: clinicopathological characteristics including age (years), body mass index (kg/m²) based on height and weight, hemoglobin concentration (g/L), total serum protein levels (g/L), serum albumin levels (g/L), ASA grade, nutritional risk screening 2002 (NRS 2002) scores, preoperative comorbidity, previous abdominal operation and muscle mass; (2) operative date: treatment plans, surgical approach, surgical outcome, lymph node dissection, intraoperative blood loss (mL), and requirement of intraoperative blood transfusion; (3) postoperative data: postoperative complications, duration of hospital stay, hospital costs and duration of postoperative drainage tube retention. Short-term postoperative complications are categorized as grade I-IV according to the Clavien-Dindo classification system [14], with observation period extended from the completion of surgery until the day of discharge.

### Measurements of skeletal muscle mass

The identification and quantification of skeletal muscle mass were performed using a dedicated processing system (Image J 1.52v, Wayne Rasband, National institutes of Health, USA). Accurate qualification of skeletal muscle mass, a fundamental parameter for evaluating overall skeletal muscle composition, often relies on analyzing the third lumbar transverse section (L3), as it demonstrates the highest correlation with whole-body skeletal muscle mass [15].Consequently, Previous studies predominantly utilized CT scan at the L3 level for the measurement of skeletal muscle mass [16, 17]. Preoperative enhanced CT of the abdomen was used to assess the nature and extent of the lesion and routine CT of the chest was used to assess cardiopulmonary function prior to anesthesia in this hospital. However, enhanced CT is not compatible with the above image processing software and the images cannot be used for body composition analysis. In addition, the prevalence of COVID-19 has increased the prevalence of routine CT of the chest [18]. In situation where L3 measurement is unavailable, previous study has identified L2, L4, L5, L1, T12, T11 and T10 ranking as the preferred alternatives in order [19]. CT scans at the T12 level emerged as a secondary choice, as they reflect whole-body skeletal muscle mass [20].

**Fig. 1:**
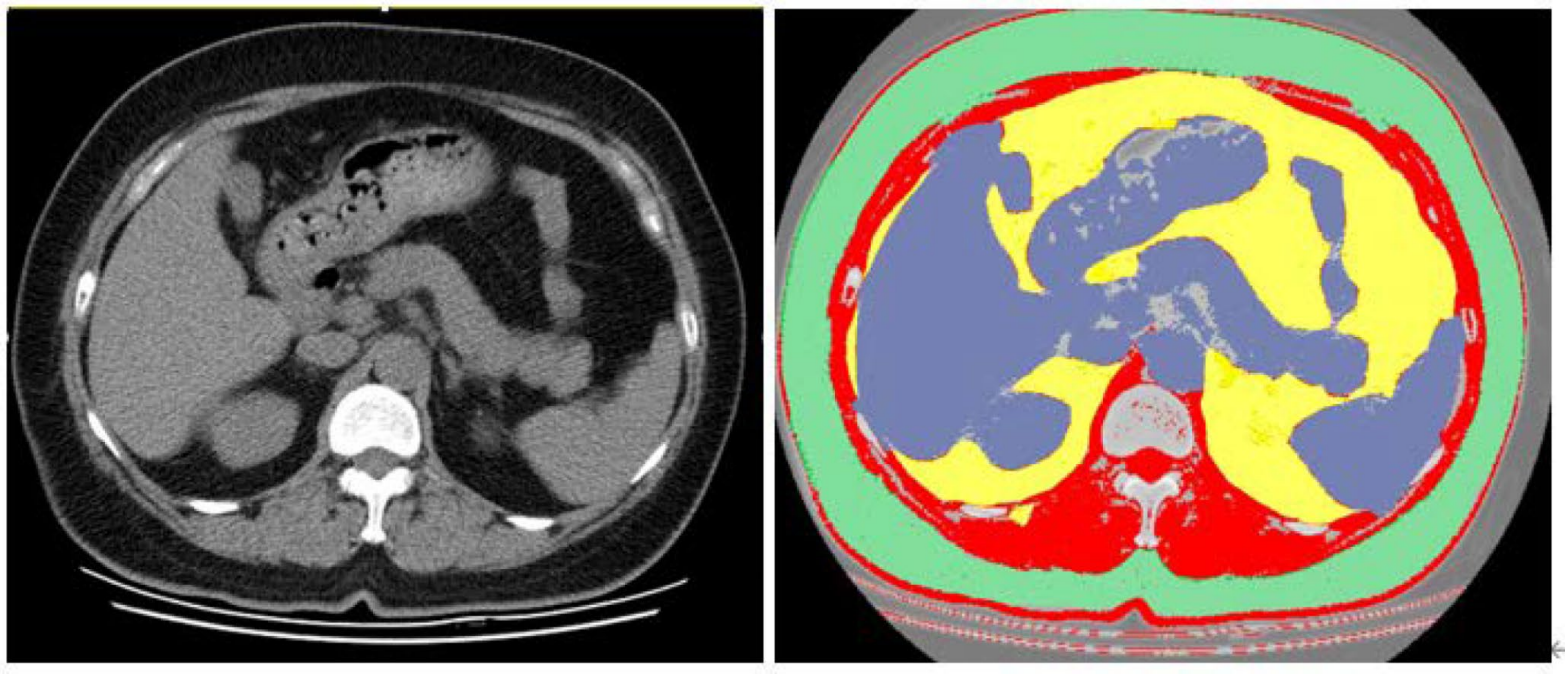
Representative segmentation of skeletal muscle area (red), visceral adipose tissue (yellow), and subcutaneous adipose tissue (green) from an axial image at the level of T12 of a CT scan. The HU values in the red part are − 29 to 150. The colored parts are quantified as areas (cm2) by the software. The skeletal muscle index was calculated as red area (cm²)/ height (m)²

A Hounsfield Unit threshold range of -29 to 150 was selected in the software to display the skeletal muscle extent in the CT images, with manual correction applied to tissue boundaries [21]. Expanding upon previous work, this study established appropriate cutoff values for low muscle mass in females: T12 skeletal muscle area (SMA) < 55.9 cm², T12 skeletal muscle index (SMI) < 20.6 cm²/m² [22]. The calculation of SMI involved dividing the total dorsal muscle of T12 cross-sectional area by the square of height (SMI = SMA/height²). An illustrative example is presented in Fig. 1.

### Statistical analysis

Statistical analysis was performed using SPSS 26.0 (IBM, Ehningen, Germany). Due to the inclusion of patients admitted using flatbeds or wheelchairs, two individual body mass index values were absent. In addition, three patients had incomplete preoperative liver function checklist records. For overall data, fewer missing values were predicted and filled using regression analysis. The normality of continuous numerical type variable was assessed using the One-Sample Kolmogrov-Smirnov tests. The comparison of continuous variables was performed using the Independent Sample T-test (or Mann-Whitney U-test), while categorical variables were compared using Pearson’s χ2 test (or Fisher’s exact test). Univariate and multivariate logistic analyses were conducted to identify underlying risk factors related to complications. *P* < 0.05 (two-tailed) was deemed statistically significant.

## Result

### Clinicopathological features of the patient

A comprehensive examination encompassing 139 patients diagnosed with ovarian cancer who met predetermined criteria were enrolled in the study between August 2017 and August 2022. The mean age of the entire cohort was 58.52 ± 11.18 years, with a median body mass index of 22.89 ± 4.13 Kg/m². Based on CT plain scan images at the T12 level, sarcopenia was identified in 24 patients with ovarian cancer, while the remaining 115 patients demonstrated normal skeletal muscle content. A thorough analysis of clinicopathological factors is listed in Table 1. As an indicator to differentiate sarcopenia, T12 skeletal muscle index was significantly lower in the sarcopenic group than in the other group (***P < 0.001). Statistical analysis revealed significantly lower body mass index (***P < 0.001) and preoperative hemoglobin level (*P = 0.017) in the sarcopenia group than the other group.

**Table 1:** Association between sarcopenia and clinical characteristics in patients with advanced epithelial ovarian cancer

Factors	Total	Sarcopenia	Non-sarcopenia	*p* value
**Age**, mean (SD), years				
	58.52(11.18)	51.42 (12.85)	60.00(10.26)	*0.001
**SMI**, median (IQR), cm² / m²				
	67.97(18.41)	51.85 (6.76)	71.3 (14.57)	*< 0.01
**BMI**, median (IQR), kg/m2				
	22.89 (4.13)	19.82 (2.27)	23.33 (3.79)	*< 0.01
**Hemoglobin**, mean (SD), g/L				
	116(21)	110.5 (11.75)	117 (22)	*0.017
**Total protein**, median (IQR), g/L				
	73.9 (8.3)	74.2 (8.68)	73.8 (8)	0.593
**Albumin**, median (IQR), g/L				
	40.6 (6.7)	40.4 (8.92)	40.7 (6.1)	0.540
**Duration of surgery** median (IQR), min				
	200(150)	255 (147.5)	190 (154)	0.093
**NRS 2002 scores**, median (IQR)				
	0.00 (1.00)	0.00 (1.00)	0.00 (1.00)	0.693
**ASA**				0.351
I/ II	131	24	107	
III/IV	8	0	8	
**Cardiopulmonary comorbidity**				0.178
No	107	21 (87.5%)	86 (74.8%)	
Yes	32	3 (12.5%)	29 (25.2%)	
**Diabetes**				1.000 (F)
No	125	22 (91.7%)	103 (89.6%)	
Yes	14	2 (8.3%)	12 (10.4%)	
**Therapy method**				
Comprehensive staged surgery	7	1	6	0.152
PDS + CT	89	19	70	
NACT + IDS	43	4	39	
**Operation method**				
Open	134	22 (91.7%)	112 (97.4%)	0.206 (F)
Laparoscopy	5	2 (8.3%)	3 (2.6%)	
**Surgical satisfaction evaluation**				
R0	100	18 (75.0%)	82 (71.3%)	0.714
R1	39	6 (25.0%)	33 (28.7%)	
R2				
**Lymph node dissection**				
Uncleaned/without metastatic	95	18 (75.0%)	77 (68.3%)	0.331
Positive pelvic lymph nodes	26	5 (20.8%)	21 (18.7%)	
Positive paraaortic lymph nodes	18	1 (4.2%)	17 (12.9%)	
**Intraoperative bleeding volume**, median (IQR), ml				
	300.00 (400.00)	300.00 (525.00)	51.00 (21.75)	0.230
**Intraoperative blood transfusion**				
No	98	16 (70.5%)	82 (71.3%)	0.650
Yes	41	8 (29.5%)	33 (28.7%)	
**Postoperative drainage tube indwelling time**,** median (IQR)**,** days**	8.00(5.00)	8.00(6.25)	8.00(5.00)	0.851
**The cost of hospitalization**,** median (IQR)**,** CNY**	41145.88( 26937.88)	41603.79(25421.16)	41145.88(27340.42)	0.493
**The length of hospital stays**,** median (IQR)**,** days**	17.00(7.50)	17.50(10.00)	17.00(7.00)	0.651
**Time to initiation of chemotherapy**, median (IQR), days	12.00(12.00)	11.50(18.75)	12.00(12.00)	0.937

*Statistically significant (*P* < 0.05). SD, standard deviation; SMI, skeletal muscle index; IQR, interquartile range; BMI, body mass index; NRS, nutritional risk screening; ASA, American Society of Anesthesiologists; PDS, primary debulking surgery; CT, chemotherapy; NACT, neoadjuvant chemotherapy; IDS, interval debulking surgery. Surgical satisfaction evaluation: R0, no visual residual tumor; R1, single residual tumor lesions with maximum longitudinal ≤ 1 cm; R2, single residual tumor lesions with maximum longitudinal>1 cm

### Effect of sarcopenia on short-term postoperative complications

The relationship between sarcopenia and postoperative outcomes is shown in Table 2. According to the Clavien-Dindo classification, A total of 65 patients experienced grade 2 or higher postoperative complications, with 16 patients belonging to the sarcopenia group and the remaining 49 patients to the normal skeletal muscle content group. It is worth mentioning that patients with more than one complication were grouped according to the highest grade of complication. Combined deep vein thrombosis of the lower extremities was the most frequently observed postoperative complication. In the group with the normal skeletal muscle content, one patient developed a postoperative intestinal anastomotic fistula that failed conservative treatment and had to be treated surgically during hospitalization. Furthermore, one patient from the sarcopenia group and four patients from the normal group were admitted to the intensive care unit (ICU) due to severe postoperative complications, including shock, organ failure, and severe systemic metabolic disorders. The rate of postoperative complications was higher in the sarcopenia group compared with the non-sarcopenia group (62.50% VS. 42.61%, *P = 0.032). However, there was no significant difference in the grade of postoperative complications between patients with sarcopenia and those without sarcopenia(*P* = 0.162). Notably, among the various postoperative complications, the incidence of intestinal obstruction requiring total parenteral nutrition was significantly higher in the sarcopenia group compared to the non-sarcopenia group, with rates of 25.0% VS. 2.61%, respectively (**P = 0.001). Additionally, while the median length of hospital stays (17.5 days VS 17.0 days) and median hospital costs (¥ 41603.79 VS. ¥ 41145.88) were slightly higher in the sarcopenia group than in the non-sarcopenia group, these differences did not attain statistical significance, the duration of drainage tube retention did not differ between the two groups.

**Table 2:** Association between sarcopenia and postoperative outcomes in patients with advanced epithelial ovarian cancer

Grade	Examples		All (*n* = 139)	Sarcopenia (*n* = 24)	Non-sarcopenia (*n* = 115)	*P*^1^ value	*P*^2^ value
**GradeI**			74(53.24%)	8(33.33%)	66(57.39%)		*0.032
**GradeII**			59(42.45%)	15(62.50%)	44(38.26%)		
	Severe anemia requiring blood transfusion		19(13.67%)	3(12.50%)	16(13.91%)	1.000	
	Intestinal obstruction requiring total parenteral nutrition		9(6.47%)	6(25.0%)	3(2.61%)	**0.001	
	Deep venous thrombosis requiring anticoagulation		32(23.02%)	6(25.0%)	26(22.61%)	0.800	
	Abdominal/ thoracic effusion requiring puncture and drainage		4(2.88%)	1(4.17%)	3(2.61%)	0.536	
	Fistula formation with conservative treatment		3(2.16%)	1(4.17%)	2(1.74%)	0.436	
	Infection requiring antibiotics		26(18.71%)	7(29.17%)	19(16.52%)	0.158	
**Grade III**			1(0.72%)	0(0%)	1(0.87%)		
	Intestinal anastomotic fistula requiring surgery		1(0.72%)	0(0%)	1(0.87%)	1.000	
**Grade IV**			5(3.60%)	1(4.17%)	4(3.48%)		
	shock		3(2.16%)	0(0%)	3(2.61%)	1.000	
	Multiple organ failure		1(0.72%)	0(0%)	1(0.87%)	1.000	
	Severe systemic metabolic disorders		1(0.72%)	1(4.17%)	0(0%)	0.173	
**Grade V**	Death		0(0%)	0(0%)	0(0%)		

* Statistically significant (*P* < 0.05). Patients who experienced more than one complication were classified as higher-grade complication

### Analysis of factors affecting postoperative complications

Univariate logistic analysis of the factors affecting the occurrence of postoperative complications was conducted to compare two groups: 65 patients with grade 2 or higher postoperative complications and 74 patients without postoperative complications. Sarcopenia (*P = 0.032), age > 65 years (**P = 0.003), albumin < 35 g/L (*P = 0.022), duration of surgery (*P = 0.019), and intraoperative bleeding volume (*P* < 0.001) were identified as factors associated with the occurrence of postoperative complications. Patients with residual lesions < 1 cm (R1) had an increased risk of postoperative complications (*P = 0.031) compared with patients with no visible residual lesions(R0). In contrast, there were no statistically difference in postoperative complications between the unsatisfactory reduction patients (R2, single residual tumor lesion > 1 cm maximum longitudinally) and patients with no visible residual lesions(R0) (*P* = 0.342) (see Table 3). No significant differences were observed among other factors, including body mass index, preoperative hemoglobin, total protein, serum albumin, NRS2002 score, ASA, preoperative cardiopulmonary disease, comorbid diabetes, history of previous abdominal surgery, treatment, surgical approach, lymph node dissection, and intraoperative blood transfusion. Through multivariate logistic analysis that controlled for potential confounding factors, sarcopenia (OR = 4.410, *P = 0.013), age over 65 years (OR = 1.063, **P = 0.002), and intraoperative bleeding volume (OR = 1.002, **P = 0.001) remained as the independent predictors for postoperative complications of ovarian cancer (Table 4).

**Table 3:** Univariate logistic analysis of factors associated with postoperative complications

		No complications (*n* = 74)	Complications (*n* = 65)	OR	95% CI	*P* value
**Age**,** years**						
	≤ 65	59	36	1		*0.003
	> 65	15	29	3.169	1.499–6.698	
**BMI**,** kg/m²**						
	< 18	3	3	1		
	18–23.9	46	39	0.848	0.162–4.442	0.845
	> 23.9	25	23	0.920	0.168–5.024	0.923
**Hemoglobin**,** g/L**						
	≥ 120	31	28	1		0.888
	< 120	43	37	0.953	0.486–1.869	
**Total protein**,** g/L**						
	≥ 65	67	55	1		0.292
	< 65	7	10	1.740	0.622–4.873	
**Albumin**,** g/L**						
	≥ 35	66	48	1		0.022*
	< 35	8	17	2.922	1.166–7.324	
**NRS-2002 score**						
	< 3	70	61	1		0.850
	≥ 3	4	4	1.148	0.275–4.785	
**ASA physical status**						
	I/II	70	61	1		0.850
	III/IV	4	4	1.148	0.275–4.785	
**Cardiopulmonary comorbidity**						
	No	53	54	1		0.113
	Yes	21	11	0.514	0.226–1.170	
**Diabetes**						
	No	67	58	1		0.798
	Yes	7	7	1.155	0.383–3.488	
**Therapy method**						
	Comprehensive staged surgery	4	3	1		
	PDS + CT	43	46	2.798	0.515–15.189	0.233
	NACT + IDS	27	16	1.481	0.257–8.547	0.660
**Operation method**						
	Open	5	0	1		0.758
	Laparoscopy	69	65	1.331	0.215–8.223	
**Surgical satisfaction evaluation**						
	R0	54	46	1		
	R1	14	9	2.909	1.101–7.689	*0.031
	R2	6	10	0.579	0.187–1.788	0.342
**Lymph node dissection**						
	Uncleaned/without metastatic	51	44	1		
	Positive pelvic lymph nodes	12	14	1.604	0.671–3.837	0.288
	Positive paraaortic lymph nodes	11	7	2.161	0.770–6.061	0.143
**Intraoperative blood transfusion**						
	No	63	35	1		0.419
	Yes	11	30	0.738	0.353–1.542	
**Duration of surgery**,** min**		180.00 (143.00)	234.00 (180.59)	1.003	1.001–1.006	0.019*
**Intraoperative bleeding volume**,** ml**		200.00 (300.00)	500.00 (600.00)	1.003	1.001–1.004	< 0.01*
**Postoperative drainage tube indwelling time**,** days**		7.00(3.50)	9.00(7.00)	1.074	0.960–1.203	0.214
**The cost of hospitalization**,** CNY**		35286.27(16782.84)	52130.74(30821.61)	1.000	1.000–1.000	0.005*
**The length of hospital stays**,** days**		16.00(5.00)	20.00(14.00)	1.046	0.975–1.122	0.212
**Time to initiation of chemotherapy**, days		9.50(10.50)	17.00(11.00)	0.998	0.989–1.007	0.645
**Sarcopenia**						
No		66	49	1		*0.032
Yes		8	16	2.694	1.067–6.798	

* Statistically significant (*P* < 0.05). OR, odds ratio; CI, confidence interval; BMI, body mass index; NRS-2002, Nutritional Risk Screening-2002; ASA, American Society of Anesthesiologists; PDS, primary debulking surgery; CT, chemotherapy; NACT, neoadjuvant chemotherapy; IDS, interval debulking surgery. Surgical satisfaction evaluation: R0, no visual residual tumor; R1, single residual tumor lesions with maximum longitudinal ≤ 1 cm; R2, single residual tumor lesions with maximum longitudinal>1 cm

**Table 4:** Multivariate logistic analysis of factors associated with postoperative complications

Factor	β	OR	95%CI	*p* value
**Sarcopenia**	1.484	4.410	1.371–14.187	*0.013
**Age**	0.061	1.063	1.022–1.105	**0.002
**Intraoperative bleeding volume**	0.002	1.002	1.001–1.004	**0.001
**Duration of surgery**	0.003	1.003	0.999–1.006	0.123
**Albumin**	0.534	1.706	0.571 − 0.096	0.339
**Surgical satisfaction evaluation**	0.117	1.125	0.461–2.744	0.796
**Constant**	-5.630	0.004		**<0.001

*Statistically significant (*P* < 0.05). β, regression coefficient; OR, odds ratio

## Discussion

### Summary of main results

Epithelial ovarian cancer (EOC) is a heterogeneous disease that varies in presentation and disease distribution. The physiological and metabolic parameters of patients with EOC can change significantly from the time of diagnosis to the entire course of treatment. Muscle mass is an indicator of metabolic and functional status and can be used as a predictive biomarker for treatment outcome [23]. The objective of this study was to explore the correlation between sarcopenia and postoperative complications in patients diagnosed with primary advanced epithelial ovarian cancer. A regression prediction model based on CT diagnosis of sarcopenia was established for the prediction of postoperative complications. While the majority of current studies are mainly concentrated on examining the correlation between sarcopenia and overall survival (OS) in ovarian cancer patients, it is crucial to consider the impact of postoperative complications on hospitalization duration, cost, and the quality of life, particularly given the short overall survival time in patients with advanced epithelial ovarian cancer. In the comparative analysis of postoperative complications performed, it was found that hospitalization costs were significantly higher in patients with postoperative complications than in patients without postoperative complications (¥ 52130.74 VS. ¥ 35286.27, *P = 0.005), which is consistent with previous studies [24]. There was a trend in the postoperative complication group towards longer drainage tube indwelling times and longer hospital stays. Although there was no statistical difference between the postoperative complication group and the non-postoperative complication group in the time to initiation of chemotherapy, there was a tendency towards longer time to initiation of chemotherapy seen in postoperative complication group which may affect disease-free survival and overall prognosis in ovarian cancer (17.00days VS 9.5days). By constructing a complication prediction model, proactive measures can be implemented to prevent and manage postoperative complications, ultimately improve the postoperative for quality of life, economic pressure and prognosis for ovarian cancer patients.

### Results in the context of published literature

Owing to the unavailability of lumbar muscles in the thoracic region covered by the chest CT scans, the dorsal muscle group of T12 was utilized as a substitute for the abdominal muscles [25]. Although the COVID-19 is gradually fading out of sight, the pulmonary sequelae caused by it and the recent ravages of Influenza A and Influenza B have led to an increase in the prevalence of chest CT and an increase in the number of people covered. Therefore, choosing chest CT rather than abdominal CT helps to detect muscle loss earlier and to intervene with the aim of minimizing the complications of abdominal surgery. Since CT scan image contain various components such as muscle, fat, and water, a Hounsfield Unit (HU) “mask” is used to exclude non-muscle pixels from the area of interest. The selection of HU value determines the identification of muscle components within the image. While Aubrey et al. defined the density range of muscles as -29 to 150 HU [21], Goodpaster et al. set the range from 0 to 100 HU [26], resulting in variation in the delineated muscle regions. In this study, the designated cut-off values for the diagnosing sarcopenia were established as skeletal muscle index < 20.6 cm²/m², coupled with a Hounsfield Unit threshold range of -29 to 150. The cutoff value was set in agreement with previous studies conducted on healthy populations [22]. Nonetheless, the type and stage of cancer, interindividual differences in muscle mass, obesity and race all have an impact on skeletal muscle index, rendering the definition of a singular “gold standard” cut-off point for sarcopenia nearly unattainable [27, 28]. In previous studies a low skeletal muscle mass was defined as a SMI < 38.50 cm2 /m2 based on recommendations of a systematic review and meta-analysis of eight studies on sarcopenia and survival in ovarian cancer [29]. The eight studies included in this analysis were selected to analyze muscle mass at the L3 or L4 level rather than the thoracic spine level. To our knowledge, no studies have reported T12 level skeletal muscle index cut-off values applicable to the diagnosis of sarcopenia in patients with advanced ovarian cancer. Therefore, our study opted to investigate the skeletal muscle index cut-off values in a healthy population, acknowledging this limitation. Future studies aim to bridge this knowledge gap.

The results of this paper are diametrically opposed to those of previous scholars, who suggested that no correlation between sarcopenia and postoperative complications [7]. The aforementioned research studied patients with intermediate to advanced (FIGO IIB-IV) ovarian cancer without the stratification of tumor pathology. The potential influence of confounding factors such as clinical stage and tumor pathology on the findings could not be adequately addressed, which may elucidate the contradictory statistical outcomes observed in this this paper. In this study, sarcopenia and elderly age (>65years) were found to be independent predictors of complications after surgery for advanced epithelial ovarian cancer. This is consistent with the findings of Vera et al. who found that low preoperative skeletal muscle density predicted poor postoperative prognosis in elderly ovarian cancer patients [30]. In contrast, that study identified age over 70 years as the advanced age group, whereas in this study it was 65 years. In addition, Ubechs et al. found that the percentage of muscle loss after two courses of neoadjuvant chemotherapy had no effect on overall survival outcomes and was associated with chemotherapy toxicity and adverse cancer-related events such as pulmonary embolism [31]. This is consistent with the results of this paper, and the study applies the concept of △SMI(the change in SMI between the scan at timepoint 1 and timepoint 2 was divided by the number of days between the scans, and subsequently multiplied by 100), which reduces bias due to individual differences and is worth promoting in future studies.

### Strengths and weaknesses

Although there are mixed findings regarding the relationship between sarcopenia and survival, the suggestion that there may be an association between them is important for patient counseling and care [32]. In elderly patients with sarcopenia, reversal of sarcopenia can be achieved with nutritional supplementation and regular physical activity, including improvements in objective strength, quality of life scores, performance of activities of daily living, and inflammatory markers [33]. The aim of this study is to diagnose sarcopenia preoperatively by CT images in order to predict the occurrence of postoperative complications, which may help guide clinicians to take appropriate measures to intervene and treat preoperative sarcopenia and prevent postoperative complications in order to achieve the goal of individualized treatment.

According to the European Working Group on Sarcopenia (EWGSOP) [34] and the Asian Working Group for Sarcopenia (AWGS) [35], the diagnosis of “sarcopenia” entails the low skeletal muscle mass, low muscle strength, and/or low physical performance. Previous studies on sarcopenia commonly relied on the combined use of CT scans, the grip strength and 6 m usual gait speed for diagnosis purpose. However, due to the non-routine measurement of grip strength and 6 m usual gait speed in the preoperative assessment of gynecological inpatients at the present hospital, couple with retrospective nature of the study resulting in the absence of data on these parameters, the sarcopenia determined in this paper should be accurately characterized as a decrease in skeletal muscle content rather than a syndrome. In a study examining the relationship between skeletal muscle reduction and tumor prognosis, Lisa Martin et al. employed skeletal muscle index as a fundamental criterion for sarcopenia diagnosis, demonstrating its potential as a predictor of tumor prognosis independent of body mass index [36]. In addition, skeletal muscle index has been utilized as a distinct diagnostic tool in studies investigating the association between sarcopenia and chemotherapy toxicity [37]. Given the employment of similar approximate estimation method by other scholars, the conclusions drawn in this study retain their informative value. Subsequent studies are encouraged to incorporate these two indicators to refine the study design. In the latest research, scholars put forward the concept of “quality is more important than quantity” and measured skeletal muscle index, skeletal muscle density, and skeletal muscle measurements using CT images. Each index was converted into Z-score to analyze its relationship with the prognosis of ovarian cancer which is worth referring in future research [38].

### Implications for practice and future research

Body mass index and various nutritional scores based on it were widely used for the assessment of nutritional status of cancer patients in clinical practice. However, in today’s society with abundant food resources, the true nutritional status of oncology patients is often masked by a normal body mass index. The diagnosis of sarcopenia with the assistance of CT images contributes to earlier prediction and intervention of postoperative complications, improving the quality of patient survival and reducing the increased costs associated with postoperative complications.

## Conclusion

This study confirms that the diagnosis of sarcopenia by CT analysis of body composition can help suggest the development of postoperative complications in advanced epithelial ovarian cancer patients and also identifies several other independent risk factors.

## Electronic supplementary material

Below is the link to the electronic supplementary material.

Supplementary Material 1

## Data Availability

No datasets were generated or analysed during the current study.

## Declarations

### Registry and the registration

N/A.

### Animal studies

N/A.

## References

[1] Siegel RL, Miller KD, Wagle NS, Jemal A (2023) Cancer statistics, 2023. CA Cancer J Clin, 73(1):17-48.10.3322/caac.21763 10.3322/caac.21763 36633525

[2] Reid BM, Permuth JB, Sellers TA (2017) Epidemiology of ovarian cancer: a review. Cancer Biol Med, 14(1):9-32.10.20892/j.issn.2095-3941.2016.0084 10.20892/j.issn.2095-3941.2016.0084 PMC5365187 28443200

[3] Lheureux S, Gourley C, Vergote I, Oza AM (2019) Epithelial ovarian cancer. Lancet, 393(10177):1240-1253.10.1016/s0140-6736(18)32552-2 10.1016/S0140-6736(18)32552-2 30910306

[4] Colombo N, Sessa C, du Bois A, Ledermann J, McCluggage WG, McNeish I, Morice P, Pignata S, Ray-Coquard I, Vergote I et al (2019) ESMO-ESGO consensus conference recommendations on ovarian cancer: pathology and molecular biology, early and advanced stages, borderline tumours and recurrent disease†. Ann Oncol, 30(5):672-705.10.1093/annonc/mdz062 10.1093/annonc/mdz062 31046081

[5] Vergote I, Leunen K, Amant F (2012) Primary surgery or neoadjuvant chemotherapy in ovarian cancer: what is the value of comparing apples with oranges? Gynecol Oncol 124(1):1–2. 10.1016/j.ygyno.2011.11.010 22153125 10.1016/j.ygyno.2011.11.010

[6] K S T, J J J, R N E, B J M, R A B (2015) Early initiation of chemotherapy following complete resection of advanced ovarian cancer associated with improved survival: NRG Oncology/Gynecologic Oncology Group study. Ann Oncol, 27.10.1093/annonc/mdv500 10.1093/annonc/mdv500 PMC4684156 26487588

[7] Rutten IJ, Ubachs J, Kruitwagen RF, van Dijk DP, Beets-Tan RG, Massuger LF, Olde Damink SW, Van Gorp T (2017) The influence of Sarcopenia on survival and surgical complications in ovarian cancer patients undergoing primary debulking surgery. Eur J Surg Oncol 43(4):717–724. 10.1016/j.ejso.2016.12.016 28159443 10.1016/j.ejso.2016.12.016

[8] Kumar A, Janco JM, Mariani A, Bakkum-Gamez JN, Langstraat CL, Weaver AL, McGree ME, Cliby WA (2016) Risk-prediction model of severe postoperative complications after primary debulking surgery for advanced ovarian cancer. Gynecol Oncol, 140(1):15-21.10.1016/j.ygyno.2015.10.025 10.1016/j.ygyno.2015.10.025 26541980

[9] van Vledder M, Levolger S, Ayez N, Verhoef C, Tran T, Ijzermans J (2012) Body composition and outcome in patients undergoing resection of colorectal liver metastases. Br J Surg 99(4):550–557. 10.1002/bjs.7823 22246799 10.1002/bjs.7823

[10] Sun X, Xu J, Chen X, Zhang W, Chen W, Zhu C, Sun J, Yang X, Wang X, Hu Y et al (2021) Sarcopenia in patients with normal body Mass Index is an independent predictor for postoperative complication and long-term survival in gastric Cancer. Clin Transl Sci 14(3):837–846. 10.1111/cts.12940 33278338 10.1111/cts.12940 PMC8212726

[11] Voron T, Tselikas L, Pietrasz D, Pigneur F, Laurent A, Compagnon P, Salloum C, Luciani A, Azoulay D (2015) Sarcopenia impacts on short- and long-term results of Hepatectomy for Hepatocellular Carcinoma. Ann Surg 261(6):1173–1183. 10.1097/sla.0000000000000743 24950264 10.1097/SLA.0000000000000743

[12] Psutka SP, Boorjian SA, Moynagh MR, Schmit GD, Costello BA, Thompson RH, Stewart-Merrill SB, Lohse CM, Cheville JC, Leibovich BC et al (2016) Decreased Skeletal Muscle Mass is Associated with an Increased Risk of Mortality after Radical Nephrectomy for Localized Renal Cell Cancer. J Urol, 195(2):270-276.10.1016/j.juro.2015.08.072 10.1016/j.juro.2015.08.072 26292038

[13] McSharry V, Mullee A, McCann L, Rogers AC, McKiernan M, Brennan DJ (2020) The impact of Sarcopenia and low muscle attenuation on overall survival in epithelial ovarian Cancer: a systematic review and Meta-analysis. Ann Surg Oncol 27(9):3553–3564. 10.1245/s10434-020-08382-0 32221737 10.1245/s10434-020-08382-0

[14] Dindo D, Demartines N, Clavien PA (2004) Classification of surgical complications: a new proposal with evaluation in a cohort of 6336 patients and results of a survey. Ann Surg, 240(2):205-213.10.1097/01.sla.0000133083.54934.ae 10.1097/01.sla.0000133083.54934.ae PMC1360123 15273542

[15] Shen W, Punyanitya M, Wang Z, Gallagher D, St-Onge MP, Albu J, Heymsfield SB, Heshka S (2004) Total body skeletal muscle and adipose tissue volumes: estimation from a single abdominal cross-sectional image. J Appl Physiol (1985) 97(6):2333–2338. 10.1152/japplphysiol.00744.2004 15310748 10.1152/japplphysiol.00744.2004

[16] Ubachs J, van de Worp W, Vaes RDW, Pasmans K, Langen RC, Meex RCR, van Bijnen A, Lambrechts S, Van Gorp T, Kruitwagen R et al (2022) Ovarian cancer ascites induces skeletal muscle wasting in vitro and reflects sarcopenia in patients. J Cachexia Sarcopenia Muscle, 13(1):311-324.10.1002/jcsm.12885 10.1002/jcsm.12885 PMC8818657 34951138

[17] Del Grande M, Rizzo S, Nicolino G, Colombo I, Rossi L, Manganaro L, Del Grande F (2021) Computed tomography-based body composition in patients with Ovarian Cancer: Association with Chemotoxicity and Prognosis. Front Oncol 11:718815. 10.3389/fonc.2021.718815 34868915 10.3389/fonc.2021.718815 PMC8634936

[18] Ebrahimzadeh S, Islam N, Dawit H, Salameh J, Kazi S, Fabiano N, Treanor L, Absi M, Ahmad F, Rooprai P et al (2022) Thoracic imaging tests for the diagnosis of COVID-19. The Cochrane database of systematic reviews, 5(5):CD013639.10.1002/14651858.CD013639.pub5 10.1002/14651858.CD013639.pub5 PMC9109458 35575286

[19] Derstine BA, Holcombe SA, Ross BE, Wang NC, Su GL, Wang SC (2018) Skeletal muscle cutoff values for Sarcopenia diagnosis using T10 to L5 measurements in a healthy US population. Sci Rep 8(1):11369. 10.1038/s41598-018-29825-5 30054580 10.1038/s41598-018-29825-5 PMC6063941

[20] Shen Y, Luo L, Fu H, Xie L, Zhang W, Lu J, Yang M (2022) Chest computed tomography-derived muscle mass and quality indicators, in-hospital outcomes, and costs in older inpatients. J Cachexia Sarcopenia Muscle 13(2):966–975. 10.1002/jcsm.12948 35178898 10.1002/jcsm.12948 PMC8977961

[21] Aubrey J, Esfandiari N, Baracos VE, Buteau FA, Frenette J, Putman CT, Mazurak VC (2014) Measurement of skeletal muscle radiation attenuation and basis of its biological variation. Acta Physiol (Oxf), 210(3):489-497.10.1111/apha.12224 10.1111/apha.12224 PMC4309522 24393306

[22] Derstine BA, Holcombe SA, Goulson RL, Ross BE, Wang NC, Sullivan JA, Su GL, Wang SC (2017) Quantifying Sarcopenia Reference Values Using Lumbar and Thoracic Muscle Areas in a Healthy Population. J Nutr Health Aging, 21(10):180-185.10.1007/s12603-017-0983-310.1007/s12603-017-0983-329300439

[23] Ataseven B, Luengo TG, du Bois A, Waltering KU, Traut A, Heitz F, Alesina PF, Prader S, Meier B, Schneider S et al (2018) Skeletal muscle attenuation (Sarcopenia) predicts reduced overall survival in patients with Advanced Epithelial Ovarian Cancer undergoing primary debulking surgery. Ann Surg Oncol 25(11):3372–3379. 10.1245/s10434-018-6683-3 30069659 10.1245/s10434-018-6683-3

[24] Ge HP, Song DF, Wu P, Xu HF (2023) Impact of sarcopenia and low muscle attenuation on outcomes of ovarian cancer: a systematic review and meta-analysis. Eur Rev Med Pharmacol Sci, 27(10):4544-4562.10.26355/eurrev_202305_32461 10.26355/eurrev_202305_32461 37259736

[25] Canvasser LD, Mazurek AA, Cron DC, Terjimanian MN, Chang ET, Lee CS, Alameddine MB, Claflin J, Davis ED, Schumacher TM et al (2014) Paraspinous muscle as a predictor of surgical outcome. J Surg Res, 192(1):76-81.10.1016/j.jss.2014.05.057 10.1016/j.jss.2014.05.057 25016439

[26] Goodpaster BH, Kelley DE, Wing RR, Meier A, Thaete FL (1999) Effects of weight loss on regional fat distribution and insulin sensitivity in obesity. Diabetes, 48(4):839-847.10.2337/diabetes.48.4.839 10.2337/diabetes.48.4.839 10102702

[27] Jones A Jr., Shen W, St-Onge MP, Gallagher D, Heshka S, Wang Z, Heymsfield SB (2004) Body-composition differences between African American and white women: relation to resting energy requirements. Am J Clin Nutr, 79(5):780-786.10.1093/ajcn/79.5.780 10.1093/ajcn/79.5.780 15113715

[28] Tan LJ, Liu SL, Lei SF, Papasian CJ, Deng HW (2012) Molecular genetic studies of gene identification for sarcopenia. Hum Genet, 131(1):1-31.10.1007/s00439-011-1040-7 10.1007/s00439-011-1040-7 21706341

[29] Ubachs J, Ziemons J, Minis-Rutten IJG, Kruitwagen R, Kleijnen J, Lambrechts S, Olde Damink SWM, Rensen SS, Van Gorp T (2019) Sarcopenia and ovarian cancer survival: a systematic review and meta-analysis. J Cachexia Sarcopenia Muscle, 10(6):1165-1174.10.1002/jcsm.12468 10.1002/jcsm.12468 PMC6903439 31389674

[30] van der Zanden V, van Soolingen NJ, Viddeleer AR, Trum JW, Amant F, Mourits MJE, Portielje JEA, van den Bos F, de Kroon CD, Kagie MJ et al (2021) Low preoperative skeletal muscle density is predictive for negative postoperative outcomes in older women with ovarian cancer. Gynecol Oncol, 162(2):360-367.10.1016/j.ygyno.2021.05.039 10.1016/j.ygyno.2021.05.039 34112514

[31] Jorne U, Simone NK, Max L, Cristina F, Leigh B, Jules SL, Henk WRS, R H H IH, dH J, vdV et al (2020) No influence of sarcopenia on survival of ovarian cancer patients in a prospective validation study. Gynecol Oncol, 159.10.1016/j.ygyno.2020.09.042 10.1016/j.ygyno.2020.09.042 33019981

[32] Staley S, Tucker K, Newton M, Ertel M, Oldan J, Doherty I, West L, Zhang Y, Gehrig P (2020) Sarcopenia as a predictor of survival and chemotoxicity in patients with epithelial ovarian cancer receiving platinum and taxane-based chemotherapy. Gynecologic oncology, 156(3):695-700.10.1016/j.ygyno.2020.01.003 10.1016/j.ygyno.2020.01.003 31928805

[33] Rondanelli M, Klersy C, Terracol G, Talluri J, Maugeri R, Guido D, Faliva MA, Solerte BS, Fioravanti M, Lukaski H et al (2016) Whey protein, amino acids, and vitamin D supplementation with physical activity increases fat-free mass and strength, functionality, and quality of life and decreases inflammation in sarcopenic elderly. Am J Clin Nutr, 103(3):830-840.10.3945/ajcn.115.113357 10.3945/ajcn.115.113357 26864356

[34] Cruz-Jentoft AJ, Baeyens JP, Bauer JM, Boirie Y, Cederholm T, Landi F, Martin FC, Michel JP, Rolland Y, Schneider SM et al (2010) Sarcopenia: European consensus on definition and diagnosis: Report of the European Working Group on Sarcopenia in Older People. Age Ageing, 39(4):412-423.10.1093/ageing/afq034 10.1093/ageing/afq034 PMC2886201 20392703

[35] Chen LK, Liu LK, Woo J, Assantachai P, Auyeung TW, Bahyah KS, Chou MY, Chen LY, Hsu PS, Krairit O et al (2014) Sarcopenia in Asia: consensus report of the Asian Working Group for Sarcopenia. J Am Med Dir Assoc, 15(2):95-101.10.1016/j.jamda.2013.11.025 10.1016/j.jamda.2013.11.025 24461239

[36] Martin L, Birdsell L, Macdonald N, Reiman T, Clandinin MT, McCargar LJ, Murphy R, Ghosh S, Sawyer MB, Baracos VE (2013) Cancer cachexia in the age of obesity: skeletal muscle depletion is a powerful prognostic factor, independent of body mass index. J Clin Oncol, 31(12):1539-1547.10.1200/jco.2012.45.2722 10.1200/JCO.2012.45.2722 23530101

[37] Del Grande M, Rizzo S, Nicolino GM, Colombo I, Rossi L, Manganaro L, Del Grande F (2021) Computed Tomography-Based Body Composition in Patients With Ovarian Cancer: Association With Chemotoxicity and Prognosis. Front Oncol, 11:718815.10.3389/fonc.2021.718815 10.3389/fonc.2021.718815 PMC8634936 34868915

[38] Polen-De C, Fadadu P, Weaver A, Moynagh M, Takahashi N, Jatoi A, LeBrasseur N, McGree M, Cliby W, Kumar A (2022) Quality is more important than quantity: pre-operative sarcopenia is associated with poor survival in advanced ovarian cancer. International journal of gynecological cancer: official journal of the International Gynecological Cancer Society.10.1136/ijgc-2022-003387 10.1136/ijgc-2022-003387 35680140

