# Supplementary material for: Sarcopenia diagnosed by computed tomography predicts postoperative complications in advanced epithelial ovarian cancer
Source: Aging Clin Exp Res. 2024 Dec 27;37(1):6. doi: 10.1007/s40520-024-02901-9 (PMC11671555; doi:10.1007/s40520-024-02901-9)
Supplement: Supplementary file 1 — Supplementary Material 1 [file 40520_2024_2901_MOESM1_ESM.pdf]

温州医科大学附属第一医院临床研究伦理委员会审查批件

(Review of Ethics Committee in Clinical Research (ECCR) of the First Affiliated Hospital of Wenzhou Medical University)

受理编号 Acceptance Number：KY2023-R288　　批件号：临床研究伦审 Issuing Number（2023）第（R288）号

项目名称 Project	术前营养状况预测卵巢癌术后并发症(Preoperative nutritional status in predicting ovarian cancer postoperative complications)		
申办者 Applicant	温州医科大学附属第一医院	试验目的 Objective	临床科研 Clinical research
试验科室 Department	妇科		
试验项目负责人 Principal Investigator	张玉阳		
审查方式和时间 Form and Date	☐ 会议审查 Review Conference，时间：________<br>☑ 快速审查 Fast track，时间：2023 年 12 月 28 日		
审查材料 Documents for Review	1、医学临床科研项目及伦理审查申请表，v1.0 版；<br>2、临床研究方案，v1.0 版，2023.12.20；<br>3、免除受试者知情同意书；<br>4、研究者团队成员目录（职责）；<br>5、主要研究者、团队成员简历及 GCP 证书，v1.0 版；<br>6、研究者责任声明；<br>7、CRF/临床观察表，v1.0 版。		
审查意见 Comments	根据国家卫健委《涉及人的生物医学研究伦理审查办法》(2016)、WMA《赫尔辛基宣言》和 CIOMS《人体生物医学研究国际道德指南》的伦理原则，经本伦理委员会审查，**同意该项目开展**。<br>According to the Regulations and Rules of "Ethical Reviews for Biomedical Research Involving Human Subjects" (2016)the National Health Commission of PRC , "Declaration of Helsinki" of WMA, and "International Ethical Guidelines for Human Biomedical Research" of CIOMS , the project was**approved**by **ECCR.**		
主任委员/副主任委员签字 Signature of the **ECCR** Chair	陈[illegible]	签发日期 Date	2023.12.31
温州医科大学附属第一医院临床研究伦理委员会（盖章）<br>Ethics Committee in Clinical Research of the First Affiliated Hospital of Wenzhou Medical University (Seal)			
附注（Note）：<br>1. 临床研究应在批准之日起 1 年内实施，逾期未实施，本批件自行废止。临床研究过程中将接受伦理委员会的跟踪审查，审查频度为自批准之日起每 12 个月一次。（伦理委员会有权根据临床试验实际开展情况改变跟			

温州医科大学附属第一医院临床研究伦理委员会专用章

踪审查频度）

The clinical study shall be implemented within 1 year from the date of approval. If overdue, the approval for this project shall berevoked. During the implementation of clinical research, tracking review will be conducted by **ECCR**every 12 months fromthe effective date of the initial approval (the ethics committee has the right to change the frequency of trackingreview according to the actual implementation of clinical trials)

2. 请严格遵从已批准的研究方案，如果方案修改需以书面形式报告伦理委员会，经伦理委员会批准后方可执行。
   Please strictly follow the approved research protocol. Any revisions of the protocol must be reported to **ECCR** in written form. It can be conducted only after the modification was approved by **ECCR**.
3. 发生严重不良事件以及影响研究风险受益比的非预期不良事件，须在 24 小时内报告本伦理委员会。
   Serious adverse events and unanticipated adverse events that affect the risk-to-benefit ratio of the project must be reported to **ECCR** within 24 hours.
4. 暂停、方案违背或提前终止临床研究，请及时上报本伦理委员会。
   Anysuspension, project violation or early termination of the clinical research, should be reported to **ECCR** promptly.
5. 完成临床研究，须提交研究完成报告给本伦理委员会。
   Please submit a completion research report to **ECCR** after completion of the project.

伦理委员会声明：

★ 温州医科大学附属第一医院临床研究伦理委员会组成及工作程序遵循中国 GCP、ICH-GCP 及相关法律法规，其审查过程不受伦理委员会以外任何组织及个人影响。

★ 本伦理委员会各委员已签署保密协议，所有标准操作规程文件、机密信息、会议记录等及其副本的所有权均归伦理委员会。

地址：浙江省温州市瓯海区南白象温州医科大学附属第一医院新院区　　邮编：325000
联系电话：0577-55578055　　传真：0577-55578033　　E-mail:wyyyclinical@126.com
